# AI chatbots for health information-seeking and self-diagnosis among Saudi university students: patterns of use, predictors, attitudes, and trust

**DOI:** 10.3389/fpubh.2026.1899249

**Published:** 2026-10-08

**Authors:** Haitham Alzghaibi, Manahil Alfuraydan

**Affiliations:** 1 Department of Health Informatics, College of Applied Medical Sciences, Qassim University, Buraydah, Saudi Arabia; 2 Health Information Management and Technology Department, College of Applied Medical Sciences, King Faisal University, Al Ahsa, Saudi Arabia

**Keywords:** AI chatbots, artificial intelligence, ChatGPT, digital health literacy, health information-seeking, Saudi Arabia, Sehhaty, self-diagnosis

## Abstract

**Background:**

The diffusion of large-language-model (LLM) chatbots such as ChatGPT has introduced a new layer to the digital health information ecosystem, alongside search engines, social media, and national mobile-health (mHealth) platforms. The actual prevalence of AI chatbot-based self-diagnosis among Saudi university students, the ecosystem of tools they choose, and the factors that predict use are not well characterised.

**Objectives:**

To estimate AI chatbot use for self-diagnosis among Saudi university students, map the ecosystem of tools used, characterise attitudes and perceived superiority over traditional search, identify predictors through logistic regression, and surface qualitative themes.

**Methods:**

A cross-sectional online survey was administered to 511 Saudi university students between April and May 2024. Instruments included a binary item on prior self-diagnosis use, a multi-response item on AI tools used, an 8-item AI Chatbot Perceived Superiority scale, a 15-item AI Doctor Chatbot Attitudes and Trust scale, five multi-response items, and three open-ended items analysed thematically. Data were analysed using descriptive statistics, multivariable logistic regression, Mann–Whitney U/Kruskal-Wallis tests, tool co-use analysis, and latent class analysis. The study received IRB approval from Qassim University (No. 23-19-02).

**Results:**

ChatGPT was the dominant tool (*n* = 355), followed by Google Gemini (*n* = 197), Microsoft Copilot (*n* = 166), and the Ministry of Health’s Sehhaty AI chatbot (*n* = 154). Multivariable logistic regression identified three independent predictors: gender [female vs. male, adjusted OR = 0.32, 95% CI (0.20, 0.52), *p* < 0.001], age (per step OR = 1.24, *p* = 0.080), and specialty (Health vs. Education OR = 6.71, *p* < 0.001). AI was rated easier and faster than search engines (*M* = 3.51) and more comprehensive (M = 3.43). Privacy concerns were prominent (*M* = 3.69); 65.1% endorsed institutionally verified AI chatbots. Tool co-use analysis showed the seven AI tools were used largely independently (max phi = 0.12).

**Conclusion:**

AI doctor chatbots have already become mainstream tools for self-diagnosis among Saudi university students, with ChatGPT dominating but the Sehhaty AI chatbot already in active use. Male students and those in Health, Management, Social Science, and Engineering are substantially more likely to use AI. The strong endorsement of institutionally verified chatbots provides a clear policy signal for embedding safe, MOH-aligned LLM tools within Sehhaty under Vision 2030.

## 1 Introduction

The way young adults find, appraise, and act on health information has been reshaped twice within 5 years. First, the COVID-19 pandemic accelerated the migration of health information-seeking to digital channels and exposed populations to an unprecedented infodemic of accurate and inaccurate content circulating side by side on social media (1–4). Second, the public release of generative artificial intelligence (AI) chatbots such as ChatGPT in late 2022 introduced a new substrate for health information retrieval a conversational interface that competes with traditional search engines, social media, and official mobile-health (mHealth) platforms (5–8). Within months of release, ChatGPT moved from novelty to routine use for symptom interpretation, lifestyle questions, and preliminary triage, particularly among digitally native cohorts such as university students (9, 10).

Digital health literacy (DHL), often used interchangeably with eHealth literacy, is defined as the ability to search electronic sources for health information, appraise its quality, and apply it to health-related decisions (11, 12). DHL determines whether a person navigates the digital information environment confidently or is overwhelmed by it (13, 14). Among university students, DHL has been linked to satisfaction with online health information (15, 16) and to the ability to respond constructively to misinformation (17). Health information-seeking behaviour (HISB) (18) is now overwhelmingly online for young adults (19, 20).

Within this evolving environment, AI doctor chatbots occupy an ambiguous and rapidly evolving position. In this manuscript, the term “AI doctor chatbot” is used descriptively to denote any generative-AI conversational tool general-purpose (e.g., ChatGPT, Google Gemini) or purpose-built (e.g., Ada Health, the Sehhaty AI chatbot) that students use to discuss symptoms or health questions; it does not imply that these tools possess the diagnostic, regulatory, or clinical authority of a licensed physician. Recent reviews report that users view AI chatbots favourably for understanding symptoms, exploring treatments, and reducing the cognitive cost of search (7, 8, 21, 22), while simultaneously expressing serious concerns about privacy, accuracy, and over-reliance (23–26). Studies comparing AI chatbots with traditional search engines have produced mixed results: some find AI offers comprehensive, easy-to-read responses while traditional search outperforms AI on the quality of public-health information in specific domains (27, 28). Trust in generative AI for health information is shaped by perceived accuracy, source transparency, institutional endorsement, and privacy assurances (29–32); AI literacy is emerging as a determinant of how users engage with generative chatbots (33, 34).

Saudi Arabia is a particularly informative setting. Under Vision 2030’s Health Sector Transformation Programme, the Ministry of Health (MOH) has developed a mature suite of mHealth platforms including the Sehhaty patient-engagement app, Mawid for appointment booking, and the 937 consultation service and has introduced its own AI chatbot embedded within Sehhaty (35). Saudi adults source health information from a heterogeneous mix of online and offline channels, with notable variation in trust (36, 37), and Saudi youth report some of the highest rates of smartphone and social media use globally. Recent work on Sehhaty has identified persistent barriers around privacy concerns, usability, and trust (35) the same dimensions students raise about commercial AI chatbots suggesting the two ecosystems compete for users’ attentional and trust budgets. Saudi studies of ChatGPT among healthcare students report broadly positive attitudes (38–40), yet most have focused on academic and educational applications rather than personal health information-seeking.

*Three gaps remain*: the population-level prevalence of AI chatbot-based self-diagnosis across multidisciplinary Saudi university students has not been quantified; the ecosystem of AI tools used commercial vs. MOH-provided has not been mapped; and the determinants of AI use, including demographic predictors and the qualitative texture of student concerns, remain underexplored.

### 1.1 Theoretical framework

This study is guided by the Digital Health AI Adoption (DHAIA) framework, an integrative model combining four traditions: eHealth literacy (11, 12), the Technology Acceptance Model and UTAUT2 (41, 42), trust transfer theory (43, 44), and HISB models (18, 45). DHAIA organises AI chatbot adoption into three layers: antecedents (socio-demographic exposure, digital health and AI literacy, HISB activation, ecosystem and institutional context, cultural context), mechanisms (perceived utility, trust calculus, institutional trust transfer), and outcomes (chatbot use, ecosystem choice, boundary maintenance with professional care).

DHAIA was chosen because no single existing theory accommodates the present setting in which demographics rather than attitudes predict use, multiple commercial and institutional AI tools co-exist, and trust in established institutions (Saudi MOH, universities) may substitute for an unfavourable trust calculus on a novel tool. The framework is shown in Figure 1 and yields seven testable propositions (P1-P7) linking antecedents to mechanisms, mechanisms to outcomes, and mechanisms to each other; these propositions structure the present results and discussion.

**Figure 1:**
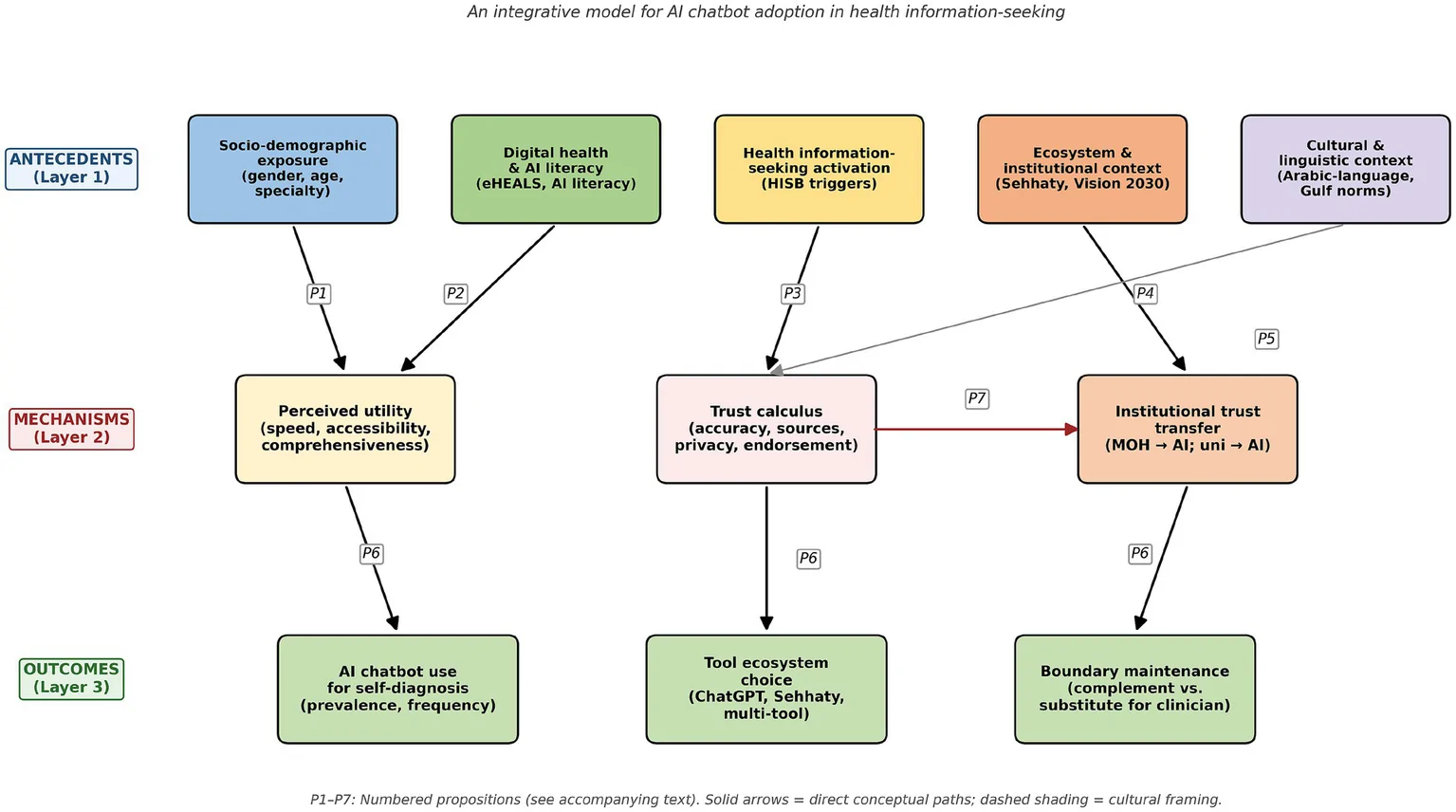
The Digital Health AI Adoption (DHAIA) framework. Antecedents (Layer 1) shape mechanisms (Layer 2), which shape outcomes (Layer 3). Propositions P1-P7 are elaborated in Section 4.

### 1.2 Study objectives

The study’s specific objectives were to: (i) estimate the prevalence of AI doctor chatbot use for self-diagnosis among Saudi university students and map the ecosystem of tools used; (ii) identify independent predictors of AI use using multivariable logistic regression; (iii) describe students’ perception of AI chatbots’ superiority over traditional search engines; (iv) characterise students’ attitudes, trust, and concerns regarding AI doctor chatbots; (v) map the principal use cases, health-issue domains, trust drivers, concerns, and preferred features; (vi) surface qualitative themes; and (vii) translate findings into actionable recommendations for embedding verified AI doctor chatbots within Saudi Arabia’s national mHealth services under Vision 2030.

## 2 Methods

### 2.1 Study design

We conducted a cross-sectional, observational online survey of Saudi university students between April and May 2024. The study followed STROBE guidelines and received ethical approval from the Institutional Review Board of Qassim University (approval number 23–19-02). All participants provided digital informed consent. Participation was voluntary, no personally identifying information was collected, and responses were stored on secure institutional infrastructure.

### 2.2 Participants and sampling

The target population comprised students currently enrolled at Saudi public universities. Inclusion criteria were: (i) age > = 18 years; (ii) current student status; (iii) ability to read Arabic or English; and (iv) provision of digital informed consent. A non-probability convenience sampling strategy was used, with the questionnaire disseminated through institutional email lists and social media platforms. The final analytical sample comprised *N* = 511 respondents. Because the survey link was circulated through open institutional email lists and social-media channels rather than sent to a fixed, enumerable list of invitees, a conventional response rate (respondents divided by invitees) could not be calculated; the implications of this for sample representativeness are discussed in Section 4.5.

### 2.3 Instrument development

The questionnaire was developed in three stages. Items were generated from prior validated scales (18, 42) and study-specific items addressing AI doctor chatbots, MOH platform familiarity, verification behaviour, and trust drivers. The instrument was translated into Arabic and back-translated into English by bilingual health-informatics specialists, then pre-tested with 15 students. The present manuscript focuses on the 8-item AI Chatbot Perceived Superiority scale, the 15-item AI Doctor Chatbot Attitudes and Trust scale, five multi-response items capturing use cases, health-issue types, trust drivers, concerns, and preferred features, and three open-ended items. All Likert items used a 5-point scale (1 = strongly disagree to 5 = strongly agree). The 8-item Perceived Superiority scale and the 15-item Attitudes and Trust scale were developed specifically for this study; although individual items drew on constructs from previously validated instruments (16, 39), neither composite scale has itself previously been validated in an Arabic-speaking population. Both scales were therefore treated as multidimensional item sets intended to capture distinct facets of use, attitude, and trust rather than as unidimensional psychometric scales with an expected single underlying factor, and were analysed descriptively at the item level rather than as summed composites. “Self-diagnosis” in this study refers specifically to the binary outcome item asking whether respondents had used an AI chatbot to interpret symptoms or reach a conclusion about a health concern; this is conceptually narrower than general health information-seeking, which also encompasses browsing for general health information without a diagnostic intent. Several of the multi-response use-case items reported in Section 3.10 (e.g., obtaining general health information, clarifying information) capture this broader information-seeking behaviour rather than self-diagnosis specifically, and the two constructs are distinguished throughout the Results and Discussion.

### 2.4 Data collection

Data were collected through an online Google Forms questionnaire administered in Arabic and English. Estimated completion time was 15–20 min. The landing page presented the participant information sheet, the IRB approval number, and the informed-consent statement. Quality-control procedures included logical-skip patterns and removal of duplicate, incomplete, or patterned responses.

### 2.5 Statistical analysis

Analyses were conducted in R version 4.3.3 and Python 3.12 (46). Continuous Likert items are reported as means and standard deviations; categorical and Likert responses as frequencies and percentages. Multi-response items were analysed by splitting comma-separated responses and visualising co-occurrence using UpSet plots.

To identify independent predictors of AI chatbot use, we estimated three multivariable logistic regression models: (1) demographic-only; (2) demographic plus 15 attitude items; (3) demographic plus full specialty contrasts. Odds ratios are reported with 95% Wald confidence intervals, alongside Wald z-statistics, McFadden pseudo R-squared, AIC/BIC, and likelihood-ratio chi-square tests. Classification accuracy at the 0.5 threshold was used as a complementary performance index.

Subgroup differences on Likert items were tested using Mann–Whitney U and Kruskal-Wallis tests, with rank-biserial correlation and epsilon-squared as effect sizes. Sampling adequacy for exploratory factor analysis was evaluated using the KMO measure and Bartlett’s test. Tool co-use was characterised by pairwise Pearson phi coefficients, Yule’s Q, and chi-square tests. Latent class analysis was conducted on 30 binary multi-response indicators using a Bernoulli mixture model with BIC-based class selection. Open-ended responses were analysed using Braun and Clarke’s six-stage inductive thematic analysis (47); coding was conducted independently by two researchers. Analysis followed all six stages: familiarisation through repeated reading of the Arabic and English free-text responses; systematic generation of initial codes; inductive collation of codes into candidate themes; review of candidate themes against the coded extracts and the full dataset; refinement and naming of themes; and final write-up with illustrative quotations (Table 1). Two researchers coded the three open-ended items independently using a shared coding framework developed after familiarisation; discrepancies in code and theme labelling were resolved through discussion and consensus, and no third adjudicator was required. Quotations originally provided in Arabic were translated into English by a bilingual member of the research team for reporting; coding was conducted on the original-language responses to preserve nuance. Given the descriptive, triangulating role of the qualitative component in this mixed-methods design, thematic saturation was assessed informally by the coding team rather than through a pre-specified stopping rule.

**Table 1:** Qualitative themes, sub-themes, frequencies, and illustrative quotations.

Theme	Sub-theme	*n*	Illustrative quotations
Q1. Experiences/expectations using AI doctor chatbots			
Perceived usefulness	Clarifying symptoms	22	“It helps me understand what my symptoms might be related to.”
	Supporting care-seeking decisions	19	“It helps me decide whether I really need to see a doctor.”
	First point of reference	17	“I usually use it as my first step before anything else.”
	Reducing uncertainty	14	“It reassures me when the problem seems minor.”
Ease of use & accessibility	Immediate access	21	“I can use it anytime without waiting.”
	User-friendly interaction	18	“The interaction feels simple and straightforward.”
	Follow-up questions	16	“I like that I can ask follow-up questions immediately.”
	Convenience	13	“It is more convenient than searching multiple websites.”
Clarity & understandability	Simple language	20	“The language is easy to understand.”
	Step-by-step explanations	15	“It explains information step by step.”
	Clear summaries	12	“The summaries are clear and helpful.”
Emotional reassurance	Reduction of anxiety	14	“It reduces my anxiety when I am worried.”
	Feeling supported	11	“It feels like having someone guide me.”
	Confidence in asking	9	“I feel more confident asking questions to AI.”
Awareness of limitations	Lack of personalisation	16	“The advice sometimes feels too general.”
	Cannot replace doctor	23	“It cannot replace a real doctor.”
	Oversimplification	10	“Some explanations feel oversimplified.”
Q2. Factors that increase/decrease trust			
Accuracy & reliability	Consistency with prof. Advice	25	“I trust it more when it matches what doctors say.”
	Evidence-based	23	“It should be based on medical evidence.”
	Avoiding contradictions	17	“Contradictory advice reduces my trust.”
Transparency	Source disclosure	18	“It should clearly state where information comes from.”
	Uncertainty acknowledgement	14	“I prefer when it admits uncertainty.”
Data privacy	Protecting personal info	24	“I worry about my personal data.”
	Secure storage	19	“I want assurance that data is stored securely.”
Institutional endorsement	Health-authority endorsement	21	“I trust it more if it is approved by health authorities.”
	Professional oversight	16	“There should be medical supervision.”
Risk of misleading advice	Incorrect advice	22	“Wrong advice could be harmful.”
	Over-reliance	15	“People should not rely on AI too much.”
Q3. Future role of AI doctor chatbots			
Supportive role	Preliminary guidance	24	“It should provide initial guidance only.”
	Referral to clinicians	22	“It should encourage consulting a doctor.”
Institutional integration	University-provided tools	20	“Universities should provide official chatbots.”
	Standardised platforms	15	“One standard platform would be helpful.”
Regulation & governance	Ethical frameworks	18	“Ethical guidelines are essential.”
	Clear boundaries	21	“Clear limits should be set.”
Education & DHL	Improving health literacy	17	“It can educate students about health.”
	Critical evaluation	13	“It should teach users how to evaluate information.”
Cautious expansion	Gradual implementation	14	“It should be introduced gradually.”
	Continuous evaluation	19	“The system should be regularly evaluated.”

## 3 Results

### 3.1 Sample characteristics

A total of 511 students completed the survey. Demographic distributions for the 505 respondents who completed all demographic items are shown in Figure 2. The sample was balanced by gender (52.2% male, *n* = 264; 47.8% female, *n* = 242) and youthful in age structure: 43.7% were 18–20 years old. Health sciences students formed the largest disciplinary group (31.4%; *n* = 159), followed by natural sciences (19.2%), management (18.6%), engineering (11.9%), social sciences and education (6.7% each), and religious sciences (5.5%).

**Figure 2:**
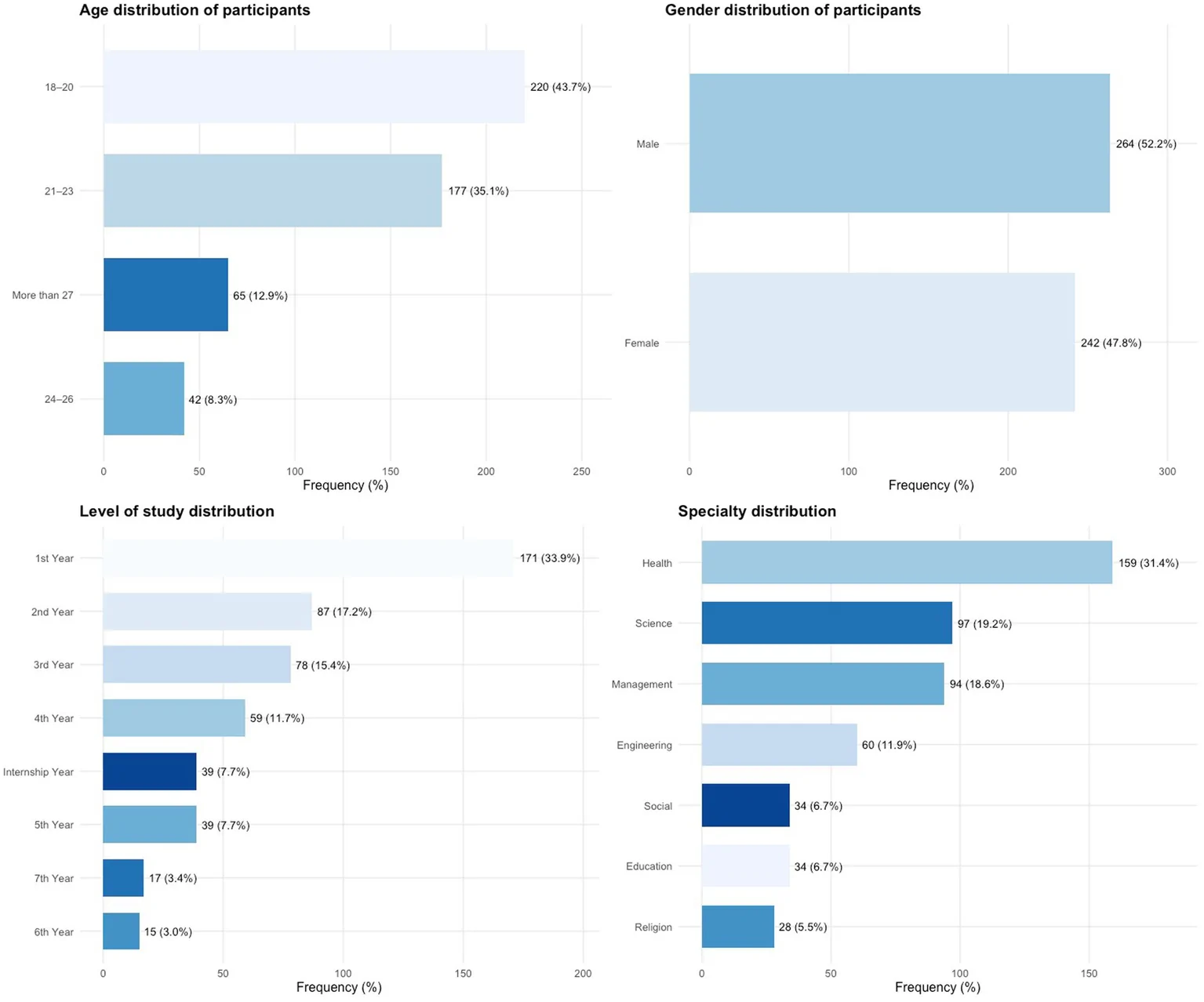
Demographic distribution of the study sample (*N* = 505).

### 3.2 Ecosystem of AI tools used for health information-seeking

The ecosystem of AI tools used for health information-seeking is visualised in Figure 3. ChatGPT was the dominant tool (*n* = 355; 93.9% of AI users), followed by Google Gemini (*n* = 197), Microsoft Copilot (*n* = 166), and the MOH-provided Sehhaty AI chatbot (*n* = 154). Perplexity AI (*n* = 68), Ada Health (*n* = 61), WebMD Symptom Checker (*n* = 53), Symptomate (*n* = 45), DeepSeek (*n* = 38), and Buoy Health (*n* = 34) were less commonly used.

**Figure 3:**
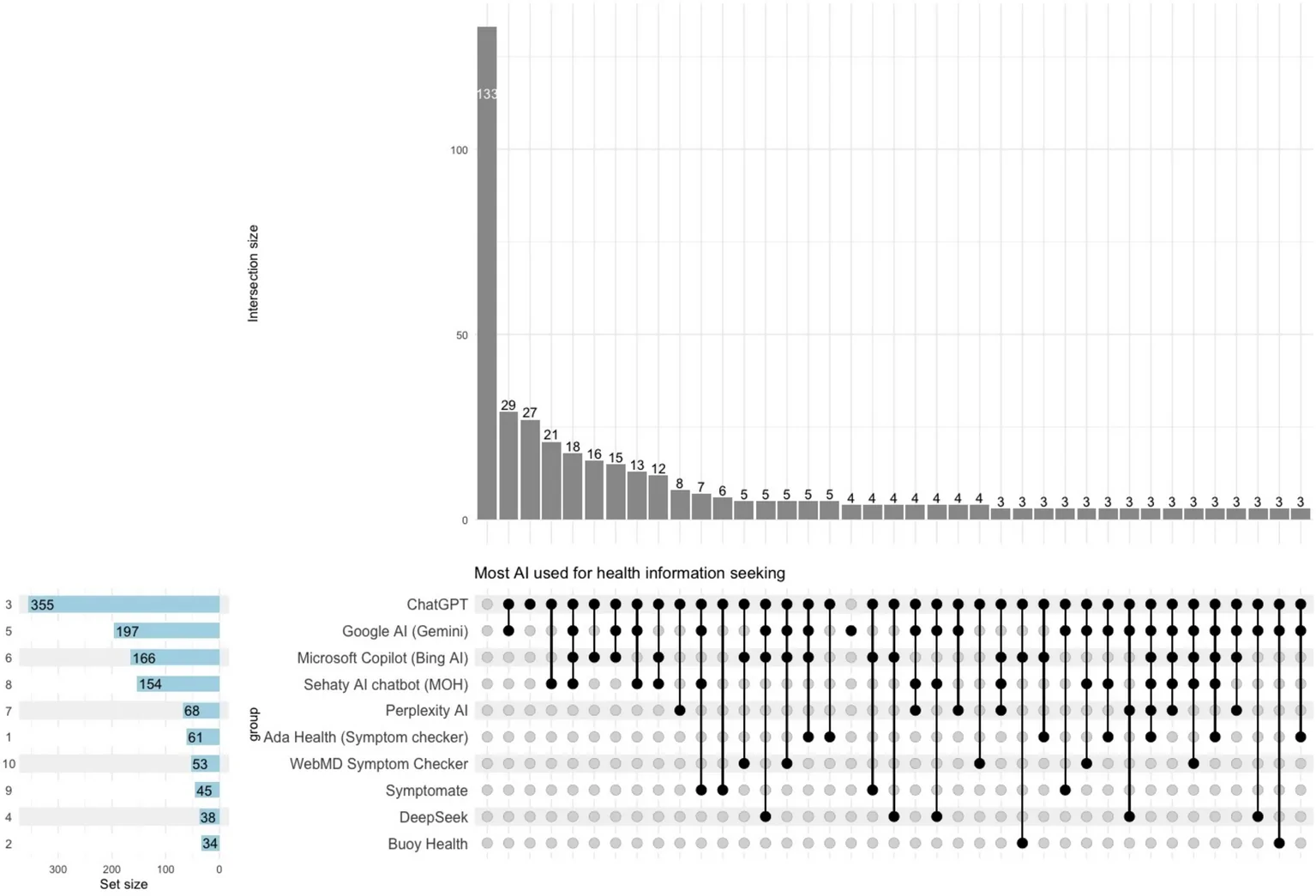
UpSet plot of AI tools used by students for health information-seeking. Left bars show total set sizes per tool; top vertical bars show intersection sizes.

Three findings stand out. First, ChatGPT functions as a near-universal default, consistent with cross-national reports (6). Second, students use multiple AI tools in combination: among the 378 AI users, mean tools per user = 2.79 (median = 3); only 11.6% used one tool exclusively (Figure 4). Third, the MOH-provided Sehhaty AI chatbot is already in active use by 40.7% of AI users.

**Figure 4:**
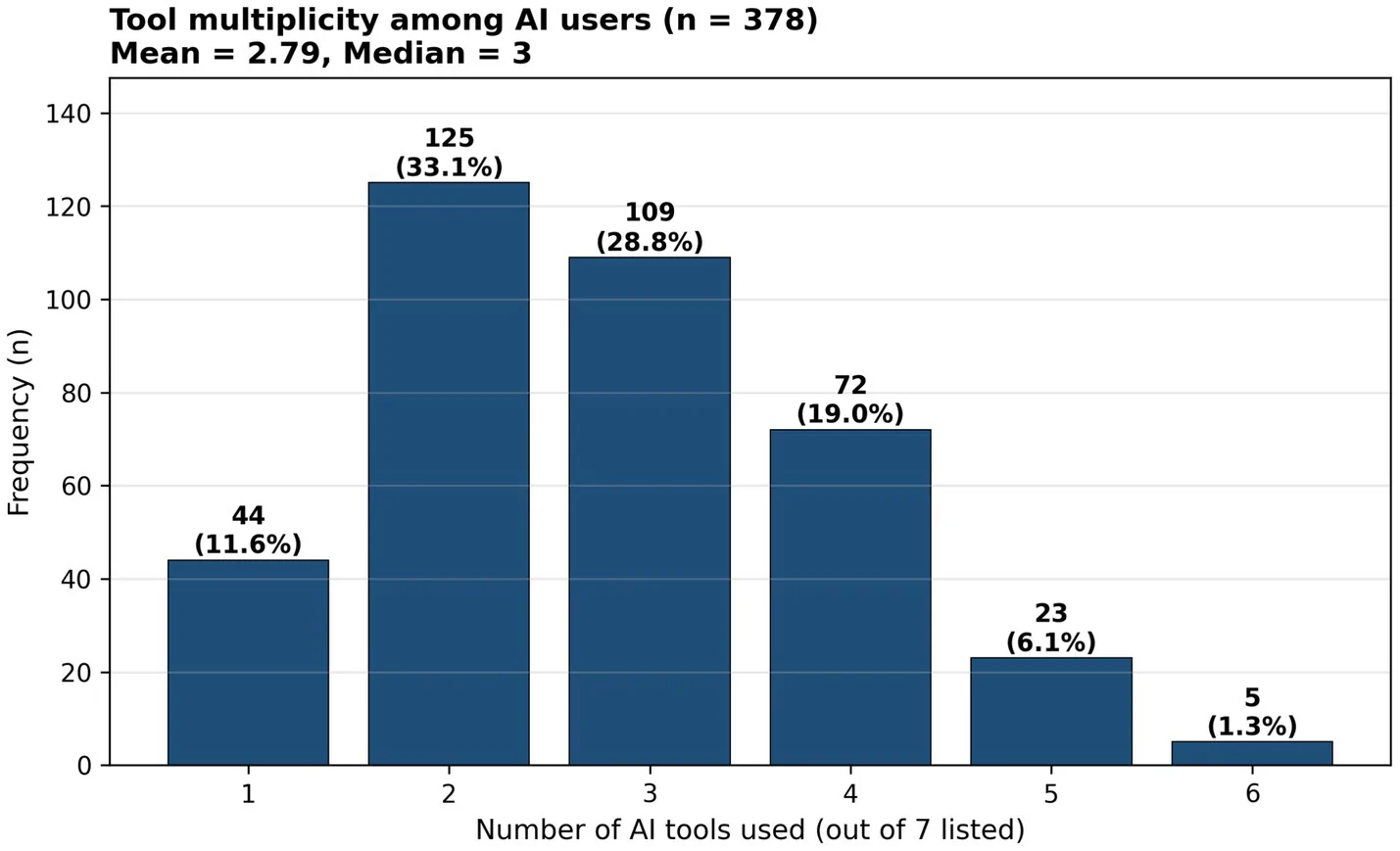
Distribution of the number of AI tools used per AI user (*n* = 378). Mean = 2.79; median = 3.

Pairwise tool co-use analysis (Pearson’s phi, Yule’s Q, and chi-square tests across all AI-tool pairs) indicated that the seven AI tools were used largely independently, with weak and predominantly non-significant associations throughout (max phi = 0.12) (full results in Supplementary Figure S1).

### 3.3 Predictors of AI chatbot use: multivariable logistic regression

To identify independent predictors of AI chatbot use, three multivariable logistic regression models were estimated (Table 2). Model 1 (demographic-only) explained 8.9% of the variance (McFadden pseudo R-squared = 0.089). Model 2 added all 15 Likert attitude items but yielded only small incremental improvement with no significant attitude predictors, indicating that demographics were more strongly associated with AI use than attitudes in this cross-sectional sample; because the design does not permit causal inference, demographics should not be interpreted as “driving” or causing AI use. Model 3 substituted a multi-category specialty contrast and achieved the best fit (pseudo R-squared = 0.122; classification accuracy = 77.2%).

**Table 2:** Multivariable logistic regression predicting AI chatbot use for self-diagnosis (*N* = 508).

Predictor	Model 1 OR (95% CI)	*p*	Model 3 OR (95% CI)	*p*
Female (vs male)	0.28 (0.18, 0.44)	< 0.001	0.32 (0.20, 0.52)	< 0.001
Age group (per step)	1.27 (1.02, 1.59)	0.036	1.24 (0.97, 1.59)	0.080
Health (vs education)^*^	2.31 (1.41, 3.79)	0.001	6.71 (2.87, 15.70)	< 0.001
Management (vs education)	—	—	5.84 (2.32, 14.72)	< 0.001
Social Science (vs education)	—	—	4.68 (1.61, 13.60)	0.005
Engineering (vs education)	—	—	3.63 (1.27, 10.39)	0.016
Sciences (vs education)	—	—	2.38 (0.99, 5.71)	0.052
Religious Sci. (vs education)	—	—	1.91 (0.66, 5.56)	0.234
Pseudo *R*-squared	0.089	—	0.122	—
Likelihood-ratio	chi^2 = 50.7, *p* < 0.001	—	chi^2 = 69.4, *p* < 0.001	—
Classification accuracy	74.2%	—	77.2%	—
AIC/BIC	536.3/553.3	—	527.3/565.4	—

*Model 1 uses a binary Health-specialty indicator; Model 3 uses full specialty contrasts with Education as reference.

Three independent predictors emerge consistently across models (Figure 5). First, gender is the strongest predictor: female students were substantially less likely than male students to have used AI for self-diagnosis [adjusted OR = 0.32, 95% CI (0.20, 0.52), *p* < 0.001]. Second, older students were slightly more likely to use AI (per-step OR = 1.24, *p* = 0.080). Third, specialty was strongly associated with use: relative to Education as the reference, students in Health [OR = 6.71, (2.87, 15.70), *p* < 0.001], Management (OR = 5.84), Social Sciences (OR = 4.68), and Engineering (OR = 3.63) were significantly more likely to use AI. None of the 15 attitudinal items reached significance in Model 2. This pattern departs from extended Technology Acceptance Model predictions (48, 49) and supports DHAIA propositions P1 and P2 (demographics shape utility; attitudes are facet-level).

**Figure 5:**
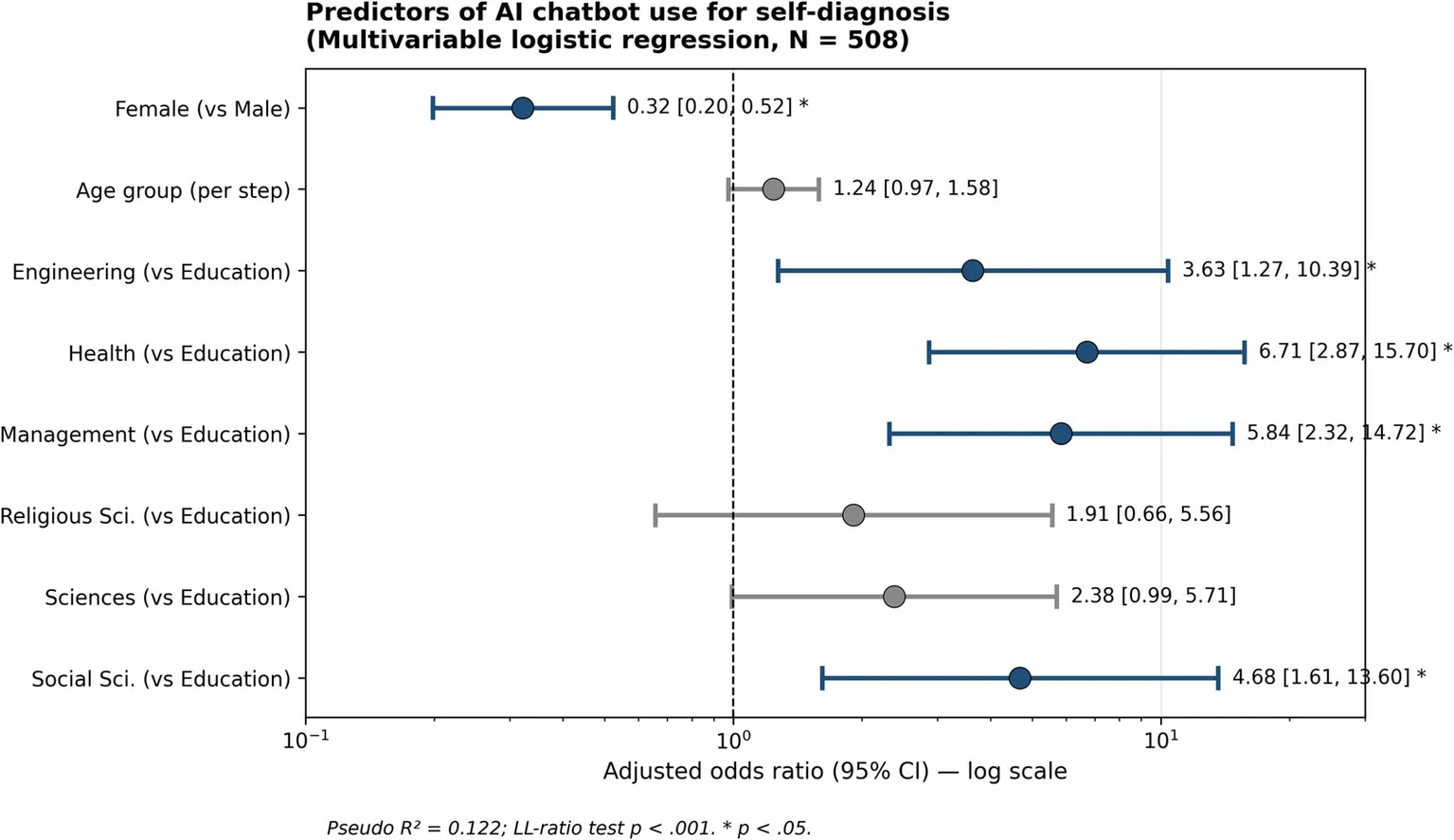
Forest plot of adjusted odds ratios from Model 3 (multivariable logistic regression; *N* = 508; pseudo *R*-squared = 0.122). blue = significant at *p* < 0.05; grey = non-significant.

### 3.4 Subgroup differences in attitudes and trust

Mann–Whitney U tests on the 15 Likert items by AI-use status revealed no significant differences (all *p* > 0.05), confirming the regression finding. By gender, one item reached significance: female students rated AI chatbots’ up-to-dateness more favourably than male students (*M* = 3.44 vs. 3.20, U = 35,798, *p* = 0.036, *r* = −0.10). Kruskal-Wallis tests by age group showed a borderline trend for safety concern (H = 7.74, *p* = 0.052). Detailed item-by-item tables are provided in Supplementary Tables S1–S4.

### 3.5 Latent class analysis

A Bernoulli-mixture latent class analysis fitted to 30 binary multi-response indicators showed BIC strongly favouring a 2-class solution. However, the 2-class solution yielded a highly unbalanced partition (Class 1 *n* = 488, 95.7%; Class 2 *n* = 22, 4.3%), and Class 2 did not differ from Class 1 on gender, AI-use status, or any Likert item. This null result indicates broad attitudinal homogeneity, suggesting universal rather than segmented policy responses are appropriate.

### 3.6 Perceived superiority of AI chatbots over traditional search engines

Students’ perceived superiority of AI chatbots over traditional search engines is summarised in Table 3. Across the eight items, means ranged from 3.09 (AI2: trust) to 3.51 (AI8: ease and speed), with all means above the neutral midpoint of 3.00. Highest endorsement was for ease and speed (56.5% combined agreement), comprehensiveness (50.6%), safety and transformative potential (52.6%), and accuracy (53.6%). Lowest endorsement was for direct preference (43.6%) and trust relative to traditional search (40.8%). These patterns echo the mixed-evidence picture in the literature (6, 27, 28): utility-oriented items attract higher endorsement than trust-oriented items, supporting the DHAIA decomposition (P6).

**Table 3:** Perceived superiority of AI chatbots over traditional search engines (*N* = 511).

Item	Statement	Mean	SD	SD %	D %	N %	A %	SA %
AI1	I prefer obtaining health information via AI chatbots compared to traditional search engines	3.13	1.42	20.9	10.7	24.7	22.1	21.5
AI2	I trust health information obtained from AI chatbots more than from traditional search engines	3.09	1.38	19.8	11.9	27.7	20.8	20.0
AI3	AI chatbots outperform traditional search engines for health information retrieval	3.34	1.37	16.6	8.3	23.5	27.5	24.1
AI4	AI chatbots are a safer and more transformative source of health information compared to traditional search	3.46	1.22	9.1	11.9	26.5	29.3	23.3
AI5	AI chatbots are my first choice for obtaining health information over traditional search engines	3.25	1.35	13.8	16.2	23.9	22.7	23.3
AI6	AI chatbots provide more accurate health information compared to traditional search engines	3.47	1.32	11.3	12.1	23.1	25.1	28.5
AI7	AI chatbots provide more comprehensive health information compared to traditional search engines	3.43	1.30	11.1	11.9	26.5	24.1	26.5
AI8	AI chatbots are easier and faster to use for health information compared to traditional search engines	3.51	1.20	8.7	11.1	23.7	33.8	22.7

SD, strongly disagree; D, disagree; N, neutral; A, agree; SA, strongly agree.

### 3.7 Attitudes, trust, and concerns regarding AI doctor chatbots

Students’ attitudes towards AI doctor chatbots are summarised in Table 4. Across the 15 items, mean endorsement ranged from 2.83 (exclusive reliance on AI for minor issues only) to 3.77 (saving time). Four meaningful clusters emerge: usefulness items drew the highest endorsement (saves time *M* = 3.77; understands symptoms *M* = 3.72; preliminary guidance *M* = 3.69), consistent with recent evidence (21, 22, 50). Trust-related items received moderate endorsement (M = 3.32–3.38) with large neutral middles (28–33%), mirroring the trust gap reported in cross-national experiments (29, 30). Concern-oriented items were strongly endorsed (privacy *M* = 3.69; 60.5% agreement), consistent with patient-population surveys (25, 31). Crucially, 65.1% agreed that universities or health institutions should provide students with access to verified AI doctor chatbots (*M* = 3.71), supporting DHAIA proposition P4 (institutional trust transfer). Two items captured the limits of AI substitution and received the lowest endorsement: exclusive reliance on AI for minor issues (*M* = 2.83) and comfort discussing sensitive issues with AI (*M* = 2.87).

**Table 4:** Attitudes, trust, and concerns regarding AI doctor chatbots (*N* = 511).

Item	Mean	SD	SD *n* (%)	D *n* (%)	N *n* (%)	A *n* (%)	SA *n* (%)
Using an AI-based doctor chatbot helps me better understand my health symptoms	3.72	1.19	31 (6.1)	58 (11.4)	92 (18.0)	171 (33.5)	159 (31.1)
AI doctor chatbots provide useful preliminary guidance before consulting a healthcare professional	3.69	1.12	25 (4.9)	54 (10.6)	112 (21.9)	182 (35.6)	138 (27.0)
Using a doctor chatbot saves time when seeking basic health information	3.77	1.16	30 (5.9)	48 (9.4)	95 (18.6)	177 (34.6)	161 (31.5)
I trust the health information provided by AI-based doctor chatbots	3.33	1.18	41 (8.0)	83 (16.2)	145 (28.4)	148 (29.0)	94 (18.4)
I believe AI doctor chatbots provide accurate health information	3.38	1.20	44 (8.6)	68 (13.3)	155 (30.3)	136 (26.6)	108 (21.1)
I am confident that AI doctor chatbots use reliable and up-to-date medical knowledge	3.32	1.20	53 (10.4)	59 (11.5)	168 (32.9)	135 (26.4)	96 (18.8)
I would rely on an AI doctor chatbot only for minor or non-urgent health issues	2.83	1.15	72 (14.1)	133 (26.0)	156 (30.5)	110 (21.5)	40 (7.8)
I am concerned about the safety of following health advice provided by AI chatbots	3.67	1.12	26 (5.1)	45 (8.8)	145 (28.4)	152 (29.7)	143 (28.0)
AI doctor chatbots should always recommend consulting a healthcare professional	3.64	1.12	25 (4.9)	59 (11.5)	119 (23.3)	181 (35.4)	127 (24.9)
I prefer consulting an AI doctor chatbot before searching for health information on general websites	3.34	1.20	40 (7.8)	80 (15.7)	163 (31.9)	120 (23.5)	108 (21.1)
AI doctor chatbots cannot replace direct consultation with a healthcare professional	3.70	1.12	21 (4.1)	54 (10.6)	129 (25.2)	158 (30.9)	149 (29.2)
I feel more comfortable discussing sensitive health issues with an AI chatbot than with a human doctor	2.87	1.22	79 (15.5)	124 (24.3)	150 (29.4)	102 (20.0)	56 (11.0)
I am concerned about the privacy of my personal health information when using AI doctor chatbots	3.69	1.14	23 (4.5)	59 (11.5)	120 (23.5)	159 (31.1)	150 (29.4)
AI doctor chatbots provided by official health authorities are more trustworthy than commercial tools	3.35	1.20	40 (7.8)	79 (15.5)	164 (32.1)	116 (22.7)	112 (21.9)
Universities or health institutions should provide students with access to verified AI doctor chatbots	3.71	1.10	24 (4.7)	53 (10.4)	101 (19.8)	204 (39.9)	129 (25.2)

### 3.8 Predictors of AI use prevalence: subgroup-specific patterns

Figure 6 illustrates the demographic patterns underpinning the regression model. Male students (84.3%) had a use rate more than 20 percentage points higher than female students (62.4%). Use prevalence increased modestly with age, from 66.8% in the 18–20 age group to 79.2–83.1% in older groups. Most striking was the specialty gradient: Management students (85.4%), Health-science students (83.0%), and Social-science students (82.4%) had use rates near or above 85%, while students in Religious Sciences (60.7%) and Education (50.0%) had use rates close to or below 60%.

**Figure 6:**
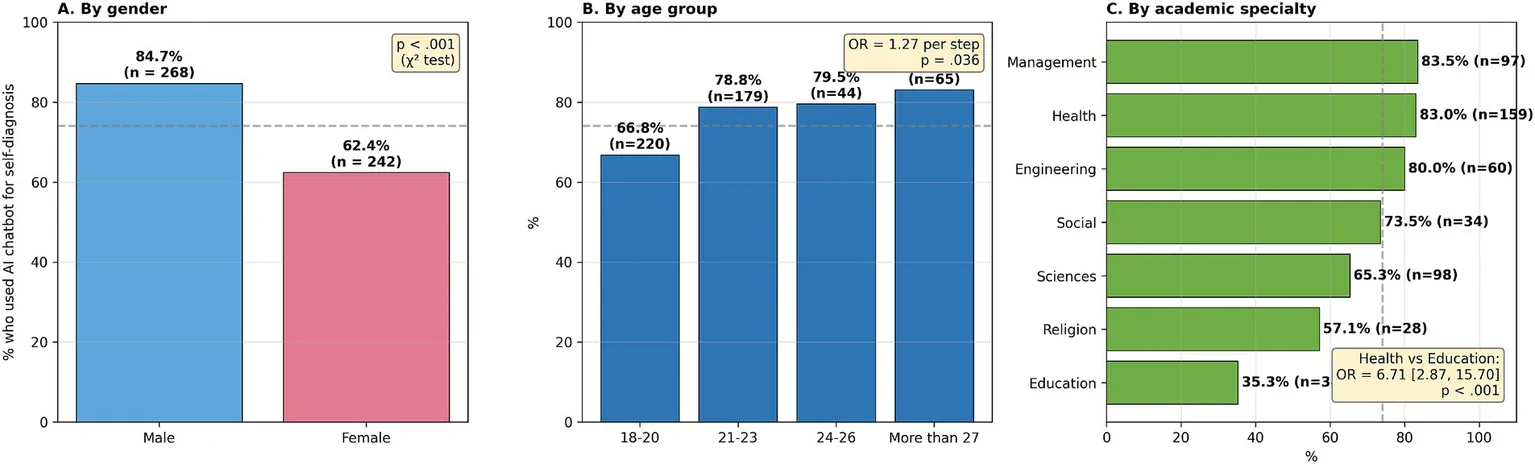
AI chatbot use prevalence by gender **(A)**, age group **(B)**, and academic specialty **(C)**. Dashed lines mark the sample-wide prevalence of 74.1%.

### 3.9 Uses, health issues, trust drivers, concerns, and features

AI doctor chatbots are used by students for a broad range of purposes (Figure 7). The most frequently reported use was obtaining general health information (*n* = 330), followed by understanding symptoms (*n* = 326). A substantial proportion also used chatbots to decide whether to see a doctor (*n* = 284), with lifestyle advice (*n* = 246) and clarifying information (*n* = 242) close behind. Preparing questions before a doctor visit (*n* = 188) was less frequent but still meaningful. Intersection patterns indicate layered, complementary use.

**Figure 7:**
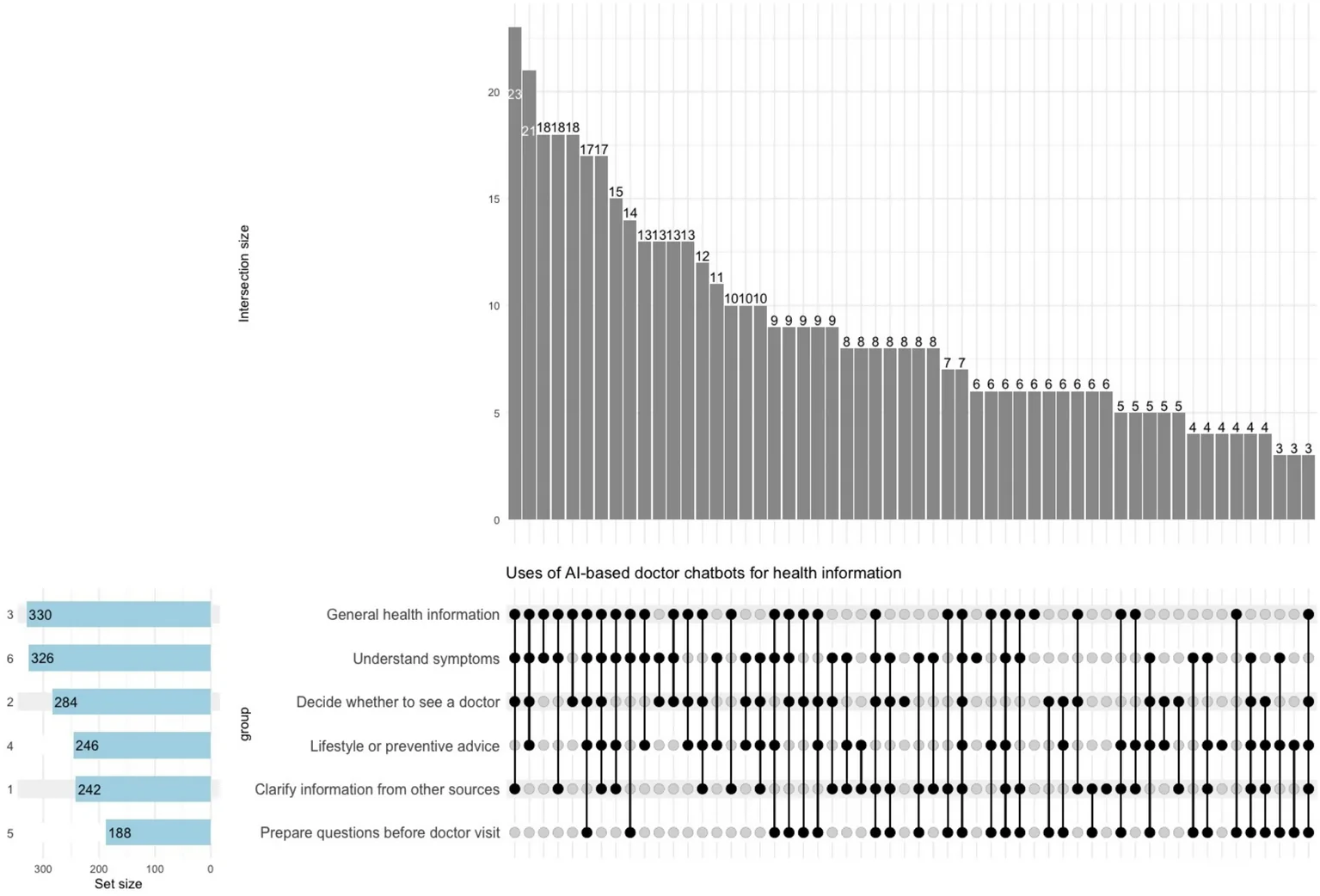
UpSet plot of uses of AI-based doctor chatbots for health information.

On health issues discussed (Figure 8), the most commonly reported category was minor or common symptoms (*n* = 349), followed by lifestyle issues (*n* = 256) and medication-related questions (*n* = 217). Mental health and wellbeing topics were reported by around 184 participants. Sensitive concerns (*n* = 147) and chronic disease management (*n* = 141) were comparatively less frequent. Students view AI chatbots as generalist tools for everyday concerns while exercising caution for more complex conditions.

**Figure 8:**
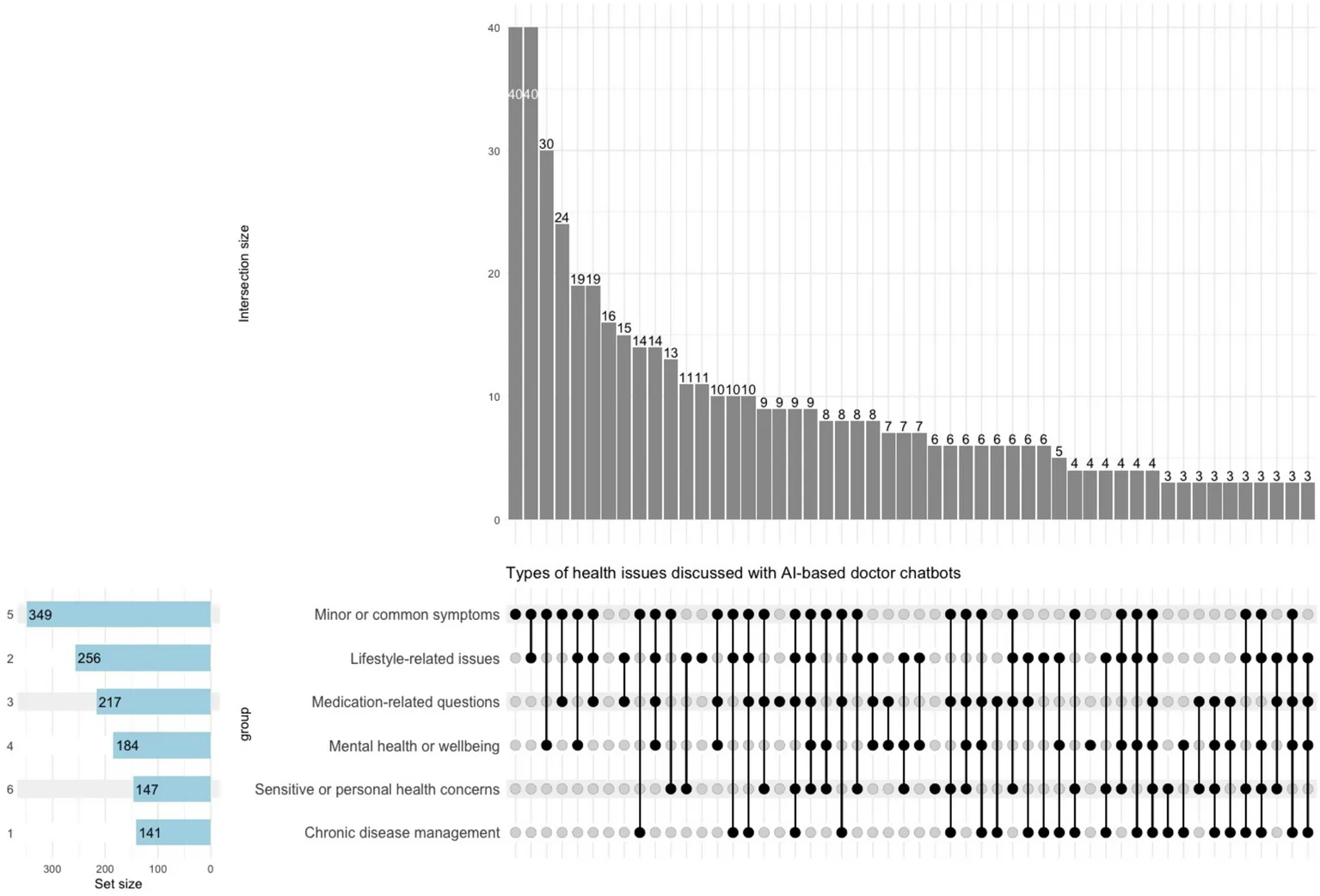
UpSet plot of types of health issues discussed with AI doctor chatbots.

Trust drivers (Figure 9) were dominated by accuracy of information (*n* = 397), clear and understandable explanations (*n* = 358), protection of personal data (*n* = 325), and recommendation to consult a doctor (*n* = 311). Transparency of data sources (*n* = 270) and endorsement by health authorities (*n* = 231) were less frequently selected.

**Figure 9:**
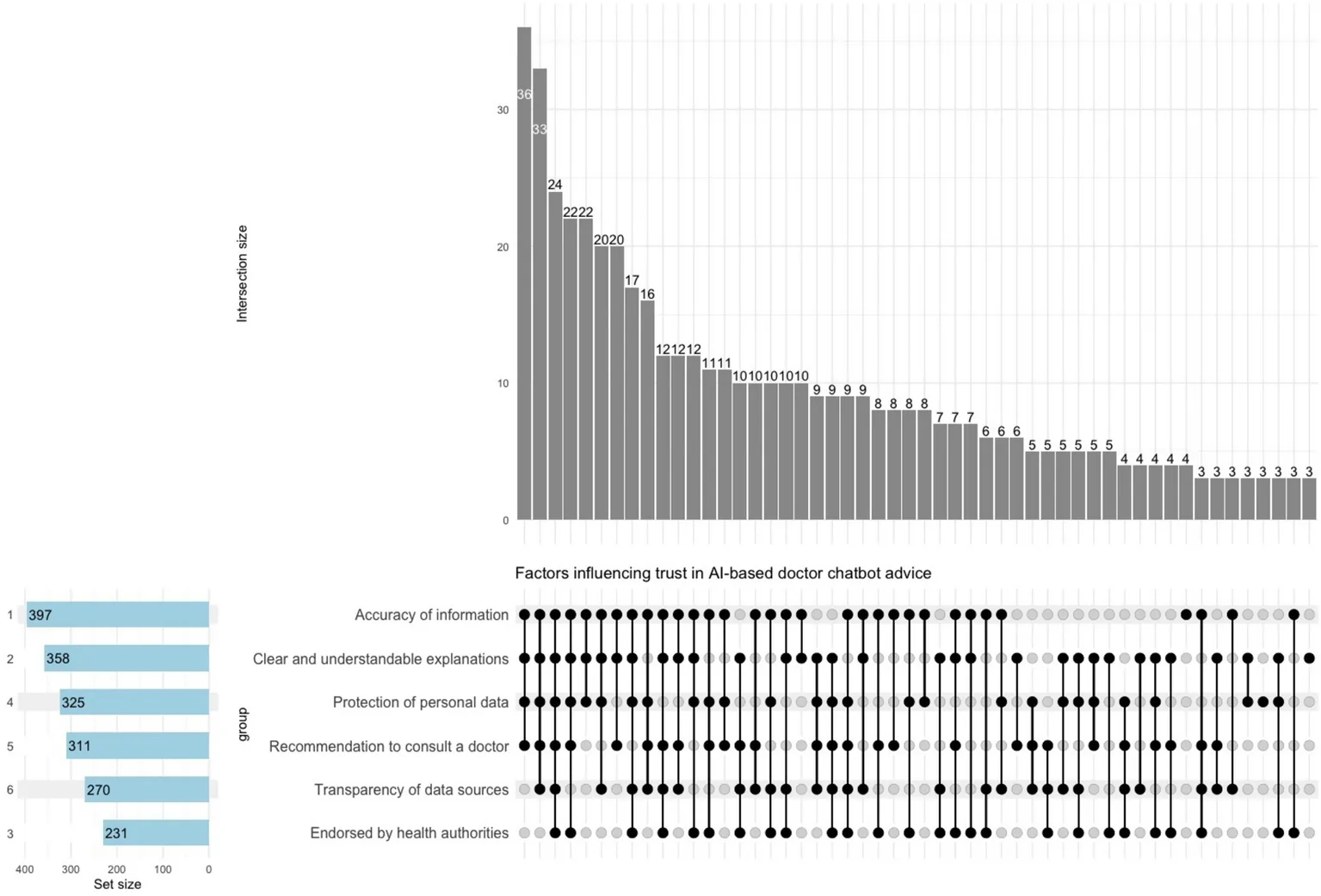
UpSet plot of factors influencing trust in AI doctor chatbot advice.

Concerns (Figure 10) were dominated by privacy and data security (*n* = 359), incorrect or misleading advice (*n* = 325), over-reliance (*n* = 275), limited understanding of complex conditions (*n* = 248), lack of accountability (*n* = 195), and ethical concerns (*n* = 173). The intersection analysis demonstrates that privacy, misinformation, and over-reliance frequently co-occur.

**Figure 10:**
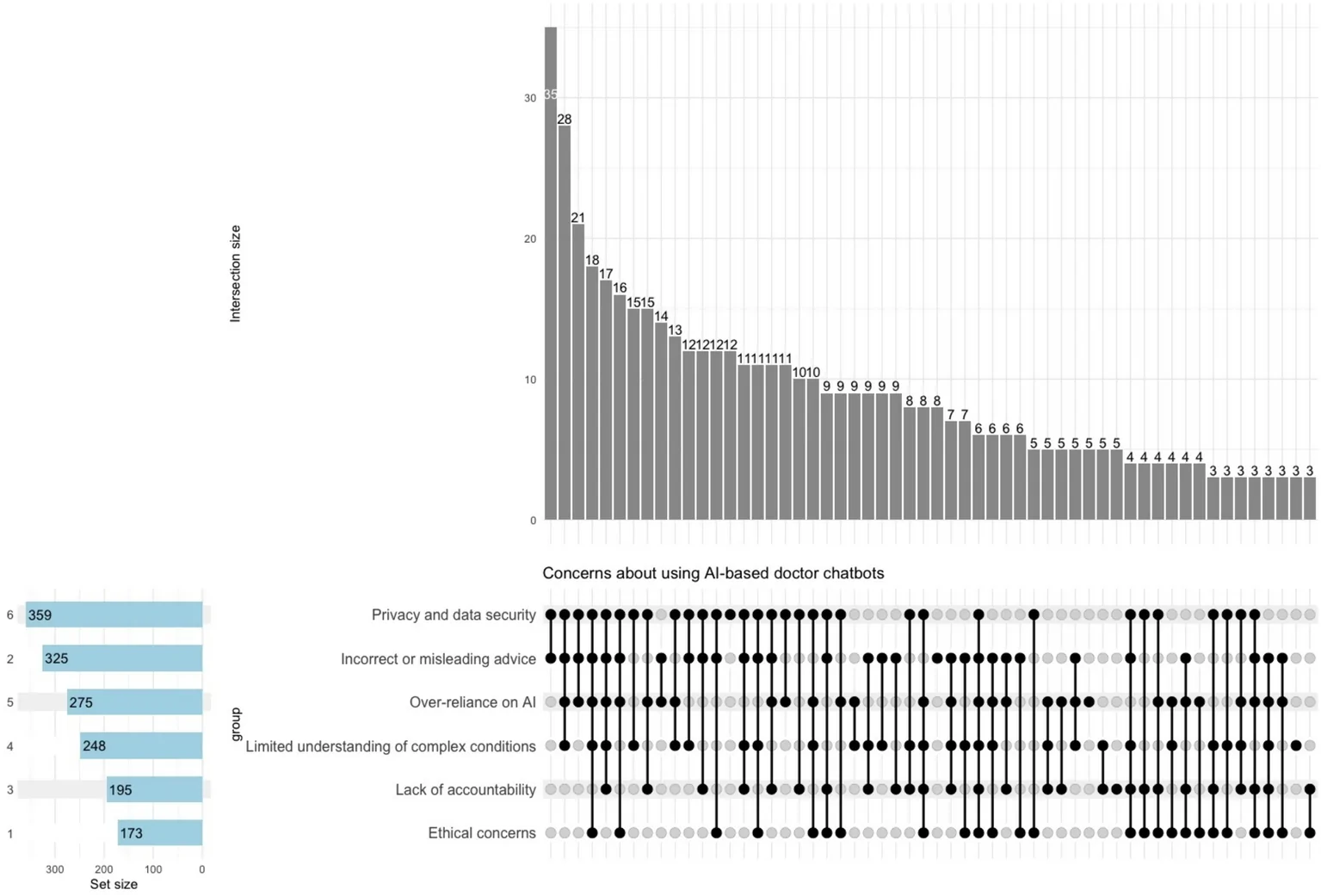
UpSet plot of concerns about using AI doctor chatbots.

Preferred features (Figure 11) emphasised clear explanations (*n* = 376), evidence-based responses (*n* = 374), ease of referral to doctors (*n* = 318), and integration with official health services (*n* = 308). Personalised advice (*n* = 266) and human-like interaction (*n* = 177) were less prioritised. Students conceptualise the ideal AI doctor chatbot as a transparent, reliable, and clinically aligned support tool.

**Figure 11:**
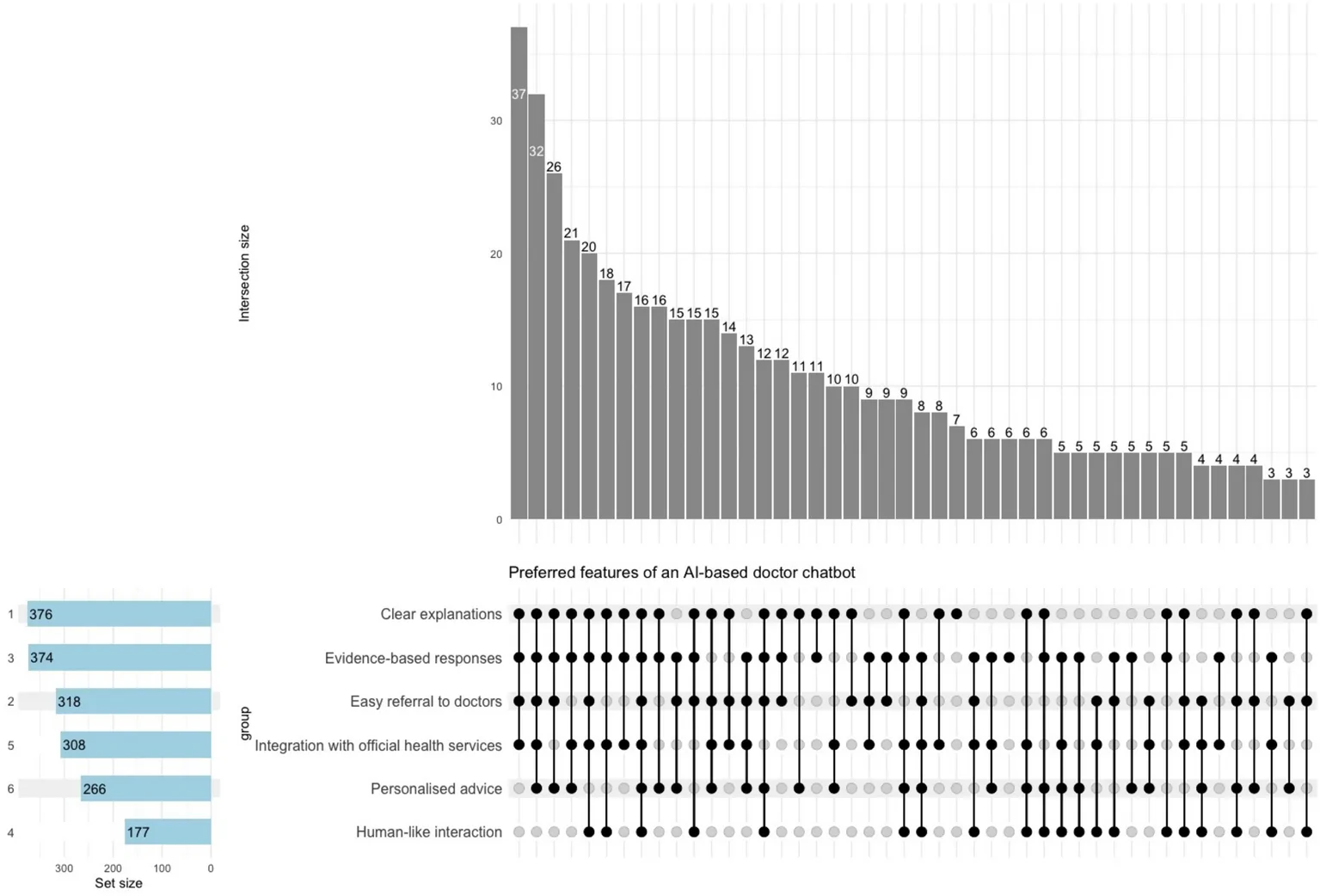
UpSet plot of preferred features of an AI doctor chatbot.

### 3.10 Qualitative themes

Inductive thematic analysis (47) of the three open-ended items produced internally consistent themes that triangulate and extend the quantitative findings (Table 1). For Q1 (experiences and expectations), five themes emerged: perceived usefulness for initial health understanding, ease of use and accessibility, clarity and understandability, emotional reassurance, and awareness of limitations. For Q2 (trust factors), five themes emerged: accuracy and reliability, transparency, privacy and confidentiality, institutional endorsement, and risk of misleading advice. For Q3 (future role), five themes emerged: supportive and complementary role, integration within institutional health services, regulation and governance, education and digital health literacy, and cautious expansion.

These qualitative themes triangulate closely with the quantitative findings above. The Q1 theme of “perceived usefulness for initial health understanding” mirrors the high-endorsement usefulness items in Table 4 (saving time, understanding symptoms), while “awareness of limitations” echoes the low endorsement of exclusive reliance on AI for minor issues (*M* = 2.83) and reinforces the boundary-maintenance interpretation developed in Section 4.2. The Q2 themes of accuracy, transparency, and institutional endorsement correspond directly to the top-ranked trust drivers in Figure 11 (accuracy of information, *n* = 397; recommendation to consult a doctor, *n* = 311) and substantively explain why trust-related Likert items remained lower and more neutrally endorsed than usefulness items: students describe trust as conditional on visible accuracy and institutional backing rather than granted by default. The Q3 themes of integration within institutional health services and regulation/governance provide the qualitative rationale behind the 65.1% quantitative endorsement of institutionally verified AI chatbots (Section 3.8), and directly inform the policy recommendations in Section 4.4. Representative quotations for each theme, together with sub-theme frequencies, are provided in Table 1.

## 4 Discussion

### 4.1 Principal findings

This cross-sectional study of 511 Saudi university students provides a structured account of how AI doctor chatbots are used, perceived, trusted, and feared. Five principal findings emerge, each mapping to a DHAIA proposition. First, AI chatbot use has crossed the mainstream threshold (74.1%) Outcome 1 from P3. Second, the ecosystem is ChatGPT-dominated but plural, with Sehhaty AI in active use by 40.7% Outcome 2 supported by P4. Third, AI chatbots are perceived as functionally superior to traditional search engines on utility dimensions while trust lags behind the utility-trust decomposition central to DHAIA Layer 2. Fourth, multivariable logistic regression identified gender, age, and academic specialty not attitudes as independent predictors of use, supporting P1 and P2. Fifth, students’ concerns and design preferences converged on a clear institutional model: 65.1% endorsed institutionally verified AI chatbots, supporting P4 and P7.

Mapping findings onto Figure 1 more precisely: P1 (socio-demographic exposure - > perceived utility/use), P2 (digital-health and AI literacy - > perceived utility), P3 (HISB activation - > use/trust calculus) and P4 (ecosystem and institutional context - > institutional trust transfer) are directly supported by the regression results, the 74.1% prevalence figure, and the 40.7% Sehhaty AI uptake reported above. P6 (utility and trust calculus - > outcomes) is partially supported by the utility-trust decomposition in Sections 3.7–3.8. P7 (trust calculus - > institutional trust transfer) is supported qualitatively by the prominence of institutional endorsement as a trust driver (Section 3.10) and the 65.1% endorsement of institutionally verified chatbots. P5 (cultural and linguistic context - > trust calculus) was not directly operationalised as a measured variable in this study (e.g., no distinct Arabic-language-use or Gulf-cultural-norms indicator was entered into the regression models) and therefore remains conceptual rather than empirically tested; we flag this explicitly as a direction for future work (Section 4.6).

### 4.2 Comparison with the literature

Our prevalence of 74.1% exceeds earlier Saudi figures focused on academic use of ChatGPT among healthcare students [66.7% positive attitudes at King Saud University (38); 94.1% usage among Umm Al-Qura medical students (39)] and extends the literature beyond healthcare specialities to a multidisciplinary sample. The dominance of ChatGPT as a near-universal default tool in our sample (93.9% of AI users) mirrors cross-national interview evidence that ChatGPT has become the default entry point for AI-assisted health information-seeking (1). It is consistent with the shifting empirical picture of online health information-seeking in young adults, where AI chatbots are increasingly integrated alongside traditional search and social media (6, 7, 10, 20).

The pattern of perceived superiority utility above trust is consistent with experimental and survey work showing that AI chatbots are experienced as faster and more comprehensible than search engines, while specific public-health information quality may remain higher in traditional sources (6, 27, 28). The strong qualitative emphasis on accuracy, transparency, and institutional endorsement as trust drivers echoes multinational evidence on what predicts intention to adopt AI-generated health information (29, 30), and the prominence of privacy concerns reflects a well-documented theme across student and patient populations (25, 31).

Two patterns in the attitude data deserve closer interpretation because they bear directly on the trust construct that is central to DHAIA and to our policy recommendations. First, the lowest-endorsed items in the Perceived Superiority scale were direct preference for AI chatbots (43.6% agreement) and trust relative to traditional search engines (40.8% agreement) both markedly lower than the utility-oriented items (ease/speed 56.5%, comprehensiveness 50.6%). This gap indicates that students find AI chatbots convenient without yet being prepared to trust or prefer them over established search tools, a dissociation consistent with the broader utility-trust decomposition described in Section 4.3 and one that limits how far utility gains alone can drive adoption of AI-based health guidance. Second, the two lowest-endorsed items on the Attitudes and Trust scale exclusive reliance on AI for minor health issues (*M* = 2.83) and comfort discussing sensitive issues with AI (*M* = 2.87) describe the outer boundary of acceptable AI use rather than general attitudes toward AI chatbots. Read together with the qualitative theme of “awareness of limitations” (Table 1), these findings suggest students conceptualise AI doctor chatbots as a bounded complement to, rather than a substitute for, professional and interpersonal care, particularly for emotionally sensitive health topics. Both patterns reinforce the practical implication in Section 4.4 that policy and design efforts should prioritise building warranted trust and clearly signalling the limits of AI guidance, rather than assuming that high utility ratings will translate into trust or substitution for clinical care.

Our finding that demographics not attitudes predict AI chatbot use is the most novel inferential contribution of this study. It departs from extended Technology Acceptance Model frameworks, which posit perceived usefulness, trust, and behavioural intention as proximal drivers (34, 48). One interpretation is that, in a digital environment where AI chatbots are ubiquitously accessible and free at the point of use, attitudes have weak predictive value because they no longer gate access; exposure-related factors dominate instead. This is consistent with the broader observation that generative AI tools are reshaping HISB in ways that bypass conventional literacy and attitudinal antecedents (10, 33).

Our documentation of an active, plural AI ecosystem in which Sehhaty’s AI chatbot already commands 40.7% of AI-user usage is theoretically and practically important. It complements work showing that Sehhaty adoption is constrained by usability, privacy, and trust concerns (35), and indicates that the perceived trust deficit of generative AI tools could be partly closed through deeper integration with existing trusted institutional platforms. The qualitative emphasis on governance, ethical frameworks, and boundaries of use is consistent with the well-documented reliability variability of current LLMs across medical domains (26, 51). The co-occurrence of privacy, misinformation, and over-reliance concerns in our intersection analysis (Figure 11) also reproduces a triad of concerns identified in recent scoping reviews of AI chatbot use for health information (3, 21).

### 4.3 Theoretical implications

Three theoretical implications follow. First, our results support a decomposed view of AI chatbot adoption in which utility perceptions and trust perceptions are largely independent dimensions, not a unitary attitude. This decomposition is consistent with extended TAM models that separate usefulness from trust antecedents (32, 48) and is reinforced by our null findings on the predictive value of attitudes once demographics are controlled. Second, our finding of a stable utility-trust gap across the entire sample suggests that interventions focused on trust formation may yield more durable adoption gains than interventions focused on perceived usefulness, which is already high. Third, the prominence of institutional endorsement as a trust driver, combined with the active uptake of Sehhaty’s AI chatbot, points to trust transfer from established institutions to AI tools (DHAIA P4 and P7) as a viable policy lever under-theorised in existing AI-acceptance frameworks but consistent with broader work on digital health literacy and trust (14, 37).

### 4.4 Practical and policy implications

Three actionable implications follow for Saudi Arabia’s Vision 2030 digital health agenda. First, the prevalence finding means policy responses must be designed for an already-engaged population. Expanding and improving the Sehhaty AI chatbot is a high-leverage intervention because it is already in active use, it addresses the demonstrated demand for institutionally trusted AI tools, and it can leverage Sehhaty’s existing user base. A redesigned Sehhaty AI chatbot should route users to professional consultation pathways, embed referral behaviour by default, use clear source disclosures, and explicitly acknowledge uncertainty features identified as critical trust drivers in our data and corroborated internationally (21, 25, 32, 50).

Second, the strong specialty gradient indicates that targeted education and policy interventions should be differentiated by disciplinary culture. Students in Education and Religious Sciences with the lowest AI use rates may benefit from baseline AI literacy training, while students in Health, Management, and Engineering already heavy users need more advanced training in source verification, hallucination recognition, and appropriate clinical referral. Embedding AI and digital health literacy in core curricula directly responds to the educational theme that emerged in our qualitative analysis (13, 14, 33).

Third, the gender disparity with females less than one-third as likely as males to use AI chatbots deserves direct attention. Whether this reflects differential access, differential confidence, differential cultural norms about technology, or genuine differences in preference cannot be determined from cross-sectional data. Targeted qualitative follow-up is needed before this gap is treated as either a problem to be closed or a preference to be respected.

### 4.5 Strengths and limitations

Strengths include a large multidisciplinary sample, an instrument covering quantitative and qualitative dimensions, multivariable logistic regression to disentangle demographic predictors, UpSet plots to visualise complex multi-response data, and structured thematic analysis. Limitations include the cross-sectional design, which precludes causal inference; the non-probability convenience sampling; the self-report nature of the survey; and the rapid evolution of the LLM landscape. Because recruitment relied on open institutional email and social-media dissemination rather than a fixed sampling frame, a conventional response rate could not be calculated, and self-selection may have favoured digitally engaged, socially connected students. Relative to published Saudi higher-education enrolment statistics, our sample over-represents Health-science students (31.4% here) and is close to gender parity, whereas national undergraduate enrolment skews female; students in smaller disciplinary groups (Religious Sciences, Education) and at universities with limited social-media outreach may be under-represented. These patterns suggest that the prevalence and predictor estimates reported here should be interpreted as characterising an engaged, multidisciplinary but not strictly representative segment of the Saudi university population, and that generalisation to the national student body should be made cautiously. The 15-item attitudes scale showed essentially no factorable structure (KMO = 0.51; Bartlett *p* = 0.67), indicating items capture distinct facets rather than a unified latent attitude. Latent class analysis on the multi-response indicators produced a 96%/4% split with no theoretically meaningful subgroup, indicating broadly homogeneous attitudes.

### 4.6 Future research directions

Three directions follow. First, longitudinal designs are needed to track whether student trust in AI doctor chatbots increases or decreases as exposure deepens. Second, experimental work directly comparing student trust and decision quality across commercial LLM chatbots, the Sehhaty AI chatbot, and traditional MOH self-assessment tools would inform integration design. Third, the strong gender and specialty gradients warrant targeted qualitative investigation of the cultural, accessibility, and confidence-related mechanisms that produce them.

## 5 Conclusion

Three out of four Saudi university students have already used AI doctor chatbots for self-diagnosis, with ChatGPT dominating the ecosystem and the MOH’s Sehhaty AI chatbot already in active use by a substantial minority. Use is independently predicted by gender (male), age (older), and academic specialty (Health, Management, Social Science, Engineering above the Education baseline) not by attitudes, which are broadly homogeneous. Students view AI chatbots as a complement to professional care rather than a substitute, expressing pronounced concerns about privacy, accuracy, and over-reliance, and demonstrating a strong preference for institutionally verified chatbots. These findings provide a clear, empirical basis for embedding safe, MOH-aligned AI doctor chatbots within Saudi Arabia’s Sehhaty ecosystem and university health services, supported by differentiated digital-health-literacy training and a governance framework consistent with Vision 2030’s digital transformation goals.

## Data Availability

The raw data supporting the conclusions of this article will be made available by the authors, without undue reservation.
