# Supplementary material for: AI chatbots for health information-seeking and self-diagnosis among Saudi university students: patterns of use, predictors, attitudes, and trust
Source: Front Public Health. 2026 Oct 8;14:1899249. doi: 10.3389/fpubh.2026.1899249 (PMC13692922; doi:10.3389/fpubh.2026.1899249)
Supplement: Supplementary file 1 [file Data_Sheet_1.PDF]

Supplementary Material

From Search Engines to AI Chatbots: Use, Attitudes, Trust, and Perceived Superiority of AI-based Doctor Chatbots for Health Information-seeking Among Saudi University Students

Supplementary Figure S1A. Full tool co-use matrices

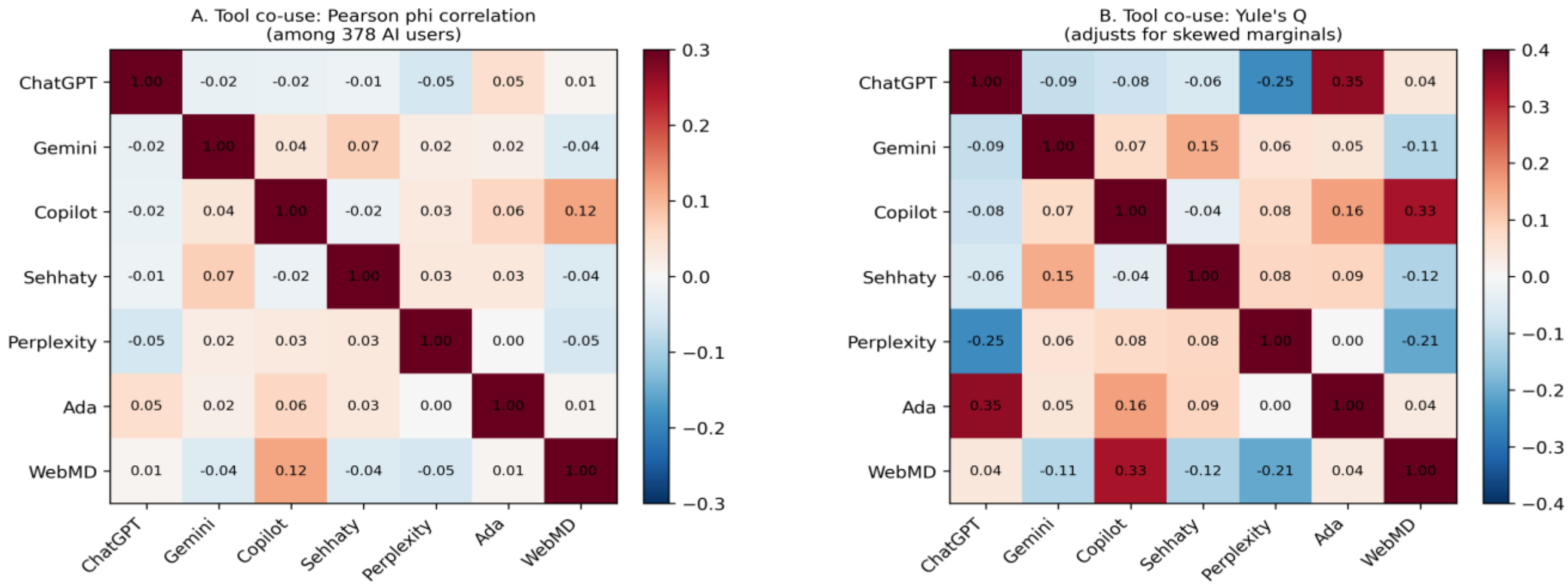

Supplementary Figure S1B. Tool co-use is uniformly weak
Larger of |Pearson phi| and |Yule's Q| per tool pair (n = 378 AI users)

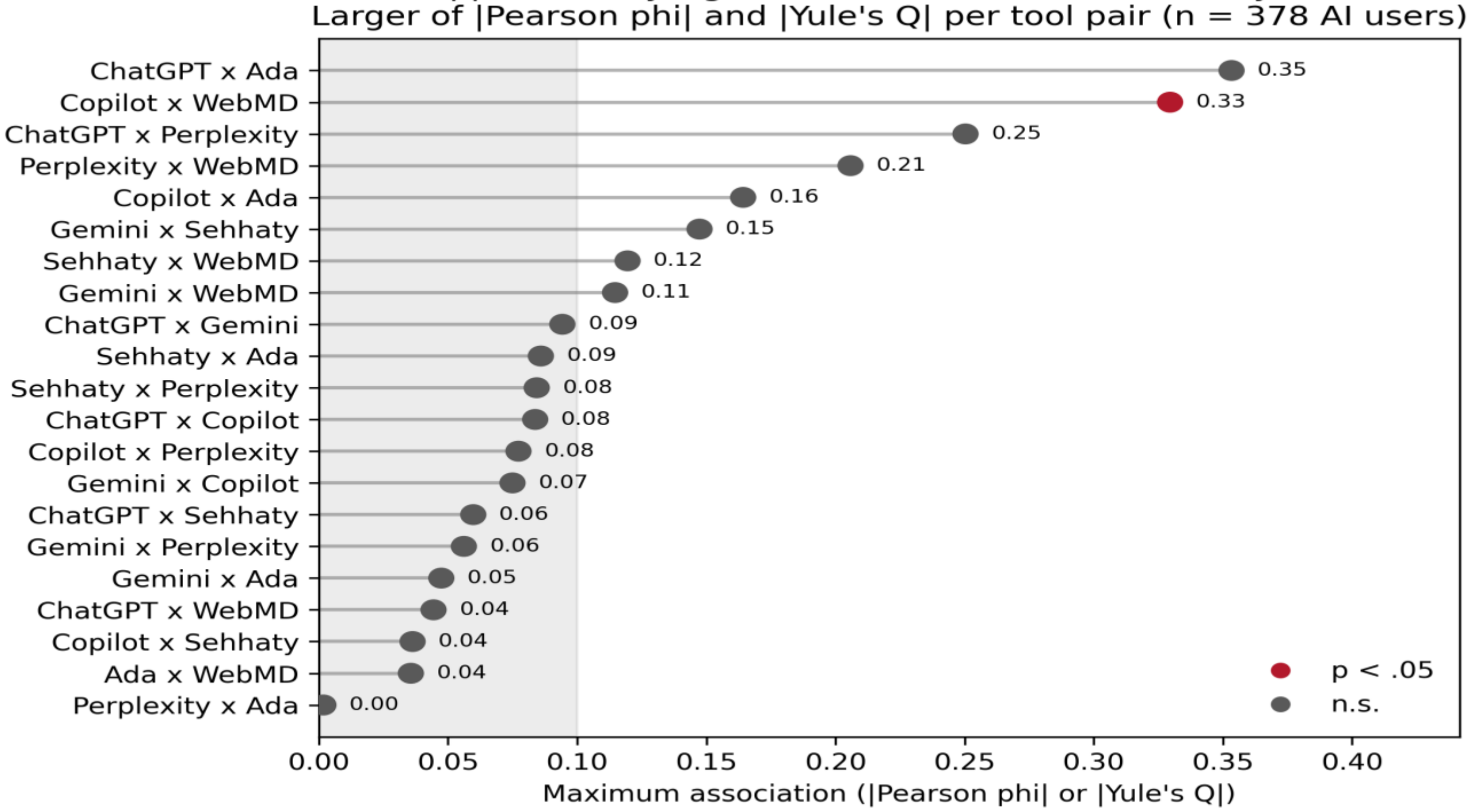

Table S1. Subgroup differences by gender (Mann-Whitney U)

Comparison of all 15 Likert items between female (n = 242) and male (n = 266) participants. Effect size r is rank-biserial correlation. *p < .05. The only statistically significant difference was for the up-to-dateness item, which female students rated slightly more favourably than males.

Item	Female M (SD)	Male M (SD)	U	p	r	Sig
**Helps understand symptoms**	3.74 (1.24)	3.7 (1.15)	33837	0.3782	-0.043	nan
**Preliminary guidance**	3.67 (1.13)	3.72 (1.1)	31597	0.6029	+0.026	nan
**Saves time**	3.69 (1.18)	3.83 (1.14)	30328	0.1876	+0.065	nan
**Trust AI information**	3.42 (1.19)	3.26 (1.18)	35157	0.0907	-0.084	nan
**AI accuracy**	3.28 (1.19)	3.47 (1.21)	29414	0.0615	+0.093	nan
**Up-to-date knowledge**	3.44 (1.17)	3.2 (1.22)	35798	0.0362	-0.104	*
**Minor issues only**	2.89 (1.18)	2.77 (1.13)	34503	0.1981	-0.064	nan
**Concern (safety)**	3.69 (1.13)	3.66 (1.12)	33044	0.7008	-0.019	nan
**Should recommend doctor**	3.7 (1.09)	3.57 (1.15)	34218	0.2630	-0.055	nan
**Prefer AI over websites**	3.38 (1.16)	3.32 (1.23)	33466	0.5200	-0.032	nan
**Cannot replace doctor**	3.66 (1.11)	3.74 (1.13)	30799	0.3092	+0.050	nan
**Comfort: sensitive issues**	2.84 (1.21)	2.89 (1.23)	31792	0.6947	+0.020	nan
**Concern (privacy)**	3.68 (1.15)	3.7 (1.14)	32072	0.8242	+0.011	nan
**Official AI more trustworthy**	3.32 (1.19)	3.39 (1.22)	31272	0.4734	+0.036	nan
**University-provided AI**	3.71 (1.07)	3.69 (1.12)	32397	0.9847	+0.001	nan

Table S2. Subgroup differences by AI-use status (Mann-Whitney U)

Comparison of all 15 Likert items between students who reported using AI doctor chatbots for self-diagnosis (Users; n = 378) and those who did not (Non-users; n = 132). No item reached statistical significance, confirming that attitudes do not differentiate users from non-users — a finding consistent with the multivariable logistic regression result that demographics, not attitudes, drive AI use.

Item	User M (SD)	Non-user M (SD)	U	p	r	Sig
**Helps understand symptoms**	3.67 (1.19)	3.86 (1.18)	22356	0.0645	+0.104	nan
**Preliminary guidance**	3.7 (1.11)	3.7 (1.14)	24688	0.8530	+0.010	nan
**Saves time**	3.78 (1.15)	3.72 (1.2)	25516	0.6850	-0.023	nan
**Trust AI information**	3.35 (1.19)	3.29 (1.18)	25631	0.6295	-0.027	nan
**AI accuracy**	3.34 (1.2)	3.49 (1.19)	23239	0.2269	+0.069	nan
**Up-to-date knowledge**	3.28 (1.22)	3.41 (1.15)	23501	0.3052	+0.058	nan
**Minor issues only**	2.83 (1.15)	2.82 (1.17)	25106	0.9113	-0.006	nan
**Concern (safety)**	3.67 (1.12)	3.67 (1.13)	25000	0.9705	-0.002	nan
**Should recommend doctor**	3.6 (1.14)	3.73 (1.07)	23387	0.2659	+0.063	nan
**Prefer AI over websites**	3.37 (1.2)	3.27 (1.2)	25910	0.4962	-0.039	nan
**Cannot replace doctor**	3.72 (1.11)	3.67 (1.15)	25425	0.7344	-0.019	nan
**Comfort: sensitive issues**	2.88 (1.23)	2.84 (1.2)	25332	0.7872	-0.015	nan
**Concern (privacy)**	3.69 (1.14)	3.68 (1.15)	25052	0.9410	-0.004	nan
**Official AI more trustworthy**	3.34 (1.21)	3.39 (1.2)	24188	0.5910	+0.030	nan
**University-provided AI**	3.69 (1.08)	3.73 (1.14)	24140	0.5614	+0.032	nan

Table S3. Subgroup differences by age group (Kruskal-Wallis)

Comparison across four age groups (18–20: n = 220; 21–23: n = 179; 24–26: n = 44; > 27: n = 65). Effect size ε^2 is epsilon-squared. *$p < .05$. Safety concern shows a borderline trend ($p = .052$) with younger students reporting slightly higher concern.

Item	18-20	21-23	24-26	> 27	H	p	ε²	Sig
**Helps understand symptoms**	3.67	3.85	3.61	3.57	5.01	0.1712	0.010	nan
**Preliminary guidance**	3.69	3.75	3.5	3.68	2.48	0.4793	0.005	nan
**Saves time**	3.79	3.75	3.82	3.71	0.28	0.9633	0.001	nan
**Trust AI information**	3.27	3.36	3.52	3.34	1.96	0.5805	0.004	nan
**AI accuracy**	3.37	3.34	3.45	3.48	0.68	0.8769	0.001	nan
**Up-to-date knowledge**	3.33	3.31	3.48	3.12	2.72	0.4373	0.005	nan
**Minor issues only**	2.81	2.92	2.61	2.82	2.23	0.5257	0.004	nan
**Concern (safety)**	3.79	3.67	3.48	3.43	7.74	0.0518	0.015	nan
**Should recommend doctor**	3.7	3.61	3.59	3.49	1.28	0.7350	0.003	nan
**Prefer AI over websites**	3.38	3.32	3.25	3.42	0.51	0.9161	0.001	nan
**Cannot replace doctor**	3.7	3.73	3.59	3.71	0.53	0.9132	0.001	nan
**Comfort: sensitive issues**	2.85	2.87	3.0	2.8	0.95	0.8122	0.002	nan
**Concern (privacy)**	3.62	3.74	3.89	3.62	3.07	0.3811	0.006	nan
**Official AI more trustworthy**	3.29	3.44	3.36	3.4	1.19	0.7556	0.002	nan
**University-provided AI**	3.67	3.65	3.68	3.95	3.71	0.2942	0.007	nan

Table S4. Subgroup differences by academic specialty (Kruskal-Wallis)

Comparison across seven academic specialties (Health: n = 159; Sciences: n = 98; Management: n = 97; Engineering: n = 60; Social: n = 34; Education: n = 34; Religion: n = 28). No Likert item reached statistical significance after multiple-testing correction.

Item	H	p	ε²	Sig
**Helps understand symptoms**	5.09	0.5328	0.010	nan
**Preliminary guidance**	5.20	0.5178	0.010	nan
**Saves time**	7.96	0.2410	0.016	nan
**Trust AI information**	6.17	0.4047	0.012	nan
**AI accuracy**	5.06	0.5357	0.010	nan
**Up-to-date knowledge**	2.85	0.8280	0.006	nan
**Minor issues only**	8.41	0.2093	0.017	nan
**Concern (safety)**	3.90	0.6906	0.008	nan
**Should recommend doctor**	8.50	0.2034	0.017	nan
**Prefer AI over websites**	11.72	0.0686	0.023	nan
**Cannot replace doctor**	1.24	0.9746	0.002	nan
**Comfort: sensitive issues**	2.71	0.8448	0.005	nan
**Concern (privacy)**	4.19	0.6510	0.008	nan
**Official AI more trustworthy**	4.45	0.6157	0.009	nan
**University-provided AI**	9.07	0.1695	0.018	nan

Table S5. Pairwise tool co-use (among 378 AI users)

Phi coefficient, Yule's Q, and Chi-square p-value for each pair of the seven most-used AI tools. Only one pair reached statistical significance: Microsoft Copilot × WebMD Symptom Checker (φ = 0.12, p = .021).

Tool A	Tool B	Both n	A only	B only	φ	Yule's Q	p
**Copilot**	WebMD	31	135	22	+0.119	+0.330	0.0211
**Gemini**	Sehhaty	87	110	67	+0.073	+0.147	0.1578
**Copilot**	Ada	31	135	30	+0.061	+0.164	0.2354
**ChatGPT**	Ada	59	296	2	+0.051	+0.353	0.3168
**Gemini**	Copilot	90	107	76	+0.037	+0.075	0.4694
**Sehhaty**	Perplexity	30	124	38	+0.032	+0.084	0.5314
**Sehhaty**	Ada	27	127	34	+0.031	+0.086	0.5410
**Copilot**	Perplexity	32	134	36	+0.030	+0.077	0.5641
**Gemini**	Perplexity	37	160	31	+0.022	+0.056	0.6757
**Gemini**	Ada	33	164	28	+0.017	+0.047	0.7351
**Ada**	WebMD	9	52	44	+0.009	+0.036	0.8571
**ChatGPT**	WebMD	50	305	3	+0.007	+0.044	0.8892
**Perplexity**	Ada	11	57	50	+0.000	+0.002	0.9923
**ChatGPT**	Sehhaty	144	211	10	-0.014	-0.060	0.7828
**Copilot**	Sehhaty	66	100	88	-0.018	-0.036	0.7310
**ChatGPT**	Copilot	155	200	11	-0.020	-0.084	0.6966
**ChatGPT**	Gemini	184	171	13	-0.022	-0.094	0.6625
**Sehhaty**	WebMD	19	135	34	-0.040	-0.120	0.4344
**Gemini**	WebMD	25	172	28	-0.040	-0.115	0.4369
**Perplexity**	WebMD	7	61	46	-0.050	-0.206	0.3283
**ChatGPT**	Perplexity	62	293	6	-0.054	-0.250	0.2968

Table S6. Latent class analysis: model selection and 2-class profile

Bernoulli-mixture latent class analysis fitted to 30 binary multi-response indicators (use cases, health issues, trust drivers, concerns, features) among the full sample of 510 valid responses, with 30 random restarts per K. The Bayesian Information Criterion (BIC) selected the 2-class solution as best. The resulting partition (Class 1: n = 488, 95.7%; Class 2: n = 22, 4.3%) did not differ on gender ($\chi^2 = 0.17$, $p = .682$), AI-use status ($\chi^2 = 0.00$, $p = 1.00$), or any of the 15 Likert items (all $p > .05$), indicating broad attitudinal homogeneity rather than meaningful latent subgroups.

Model selection

K	Log-likelihood	BIC	AIC	Entropy
**2**	-9,914.18	20,208.66 ← best	19,950.36	0.942
**3**	-9,881.79	20,337.15	19,947.58	0.826
**4**	-9,845.77	20,458.37	19,937.54	0.871
**5**	-9,813.56	20,587.22	19,935.12	0.831

Top differentiating indicators between 2 classes (Class 1 minus Class 2 probability)

Indicator	P(item|Class 1)	P(item|Class 2)	Difference
**Easy referral to doctors (feature)**	0.647	0.001	+0.646
**Clarify info from other sources (use)**	0.492	0.001	+0.491
**Endorsed by health authorities (trust)**	0.435	0.877	–0.441
**Integration with official services (feature)**	0.586	0.999	–0.413
**Personalised advice (feature)**	0.505	0.885	–0.381
**Understand symptoms (use)**	0.622	0.999	–0.377
**Privacy concerns**	0.716	0.357	+0.360
**Minor symptoms (issue)**	0.669	0.999	–0.330
**Incorrect advice concerns**	0.648	0.325	+0.323
**Clear explanations (feature)**	0.725	0.997	–0.272

Class 2 (n = 22; 4.3%) is characterised by a near-uniform endorsement of 'personalised', 'official', and 'institutionally endorsed' features combined with lower endorsement of privacy and misinformation concerns. It represents a small enthusiast minority rather than a substantively distinct attitudinal group.
